# Progress in understanding and improving oil content and quality in seeds

**DOI:** 10.3389/fpls.2023.1116894

**Published:** 2023-01-26

**Authors:** Julius Ver Sagun, Umesh Prasad Yadav, Ana Paula Alonso

**Affiliations:** Department of Biological Sciences, BioDiscovery Institute, University of North Texas, Denton, TX, United States

**Keywords:** carbon conversion efficiency, embryo culture, fatty acid synthesis, isolated plastids, lipid storage, metabolic flux analysis, oilseed, triacylglycerol

## Abstract

The world’s population is projected to increase by two billion by 2050, resulting in food and energy insecurity. Oilseed crops have been identified as key to address these challenges: they produce and store lipids in the seeds as triacylglycerols that can serve as a source of food/feed, renewable fuels, and other industrially-relevant chemicals. Therefore, improving seed oil content and composition has generated immense interest. Research efforts aiming to unravel the regulatory pathways involved in fatty acid synthesis and to identify targets for metabolic engineering have made tremendous progress. This review provides a summary of the current knowledge of oil metabolism and discusses how photochemical activity and unconventional pathways can contribute to high carbon conversion efficiency in seeds. It also highlights the importance of ^13^C-metabolic flux analysis as a tool to gain insights on the pathways that regulate oil biosynthesis in seeds. Finally, a list of key genes and regulators that have been recently targeted to enhance seed oil production are reviewed and additional possible targets in the metabolic pathways are proposed to achieve desirable oil content and quality.

## Introduction

Depending on plant species, seeds accumulate various proportions of biomass components, such as proteins, starch and lipids. In seeds, storage oils are mainly in the form of triacylglycerols (TGs), as an energy reserve utilized during germination and post-germinative growth. These oilseeds have a profound agricultural and industrial significance, utilized predominantly in food processing and preparation, and as a renewable resource for various industrial applications (Jaworski and Cahoon, 2003). Because of their structural similarity with long-chain hydrocarbons, TGs can replace petroleum-based products, such as diesel, lubricants, paints, coatings or inks (Cahoon et al., 2007; Durrett et al., 2008). The renewable biofuels derived from oilseeds produce ~85% less carcinogens during combustion than petroleum-based diesel fuels, presenting advantages in terms of sustainability, environment and health (Atadashi et al., 2012; Hasan and Rahman, 2017). Because of its popularity as a renewable resource, the consumption of seed oil has been increasing simultaneously with the rapidly growing population and upgraded standards of living (Marchive et al., 2014; Samarth and Mahanwar, 2015). To meet these rising demands, there is an urgent need to develop new oilseed cultivars with improved oil content and composition (Singer et al., 2013; Xu et al., 2018). Many research efforts have been focused into the improvement of seed oil over the years using conventional or molecular-assisted breeding approaches, as well as more targeted genetic manipulation.

The selection of candidate genes that can be engineered relies on understanding the biochemical regulations that control carbon partitioning during *de novo* fatty acid synthesis (FAS) in seeds. Biosynthesis of TGs starts from FAS in plastids which relies on a cycle of condensation, reduction and dehydration reactions that extend an acyl-chain linked to an acyl carrier protein (ACP) by two carbon units per cycle. These fatty acids (FAs) are then assembled into TGs in the endoplasmic reticulum (ER), or used in other metabolic processes, such as chain elongation and acyl editing. The carbon precursor for FAS is acetyl-CoA, which is generated from the oxidative decarboxylation of pyruvate through the pyruvate dehydrogenase complex (Johnston et al., 1997). Determining the efficiency of the developing embryo in converting this carbon source into oil and other biomass components (e.g. proteins, and carbohydrates) known as carbon conversion efficiency (CCE), is important to evaluate the potential for improving seed oil quality (Goffman et al., 2005; Alonso et al., 2007; Allen et al., 2009; Lonien and Schwender, 2009; Chen and Shachar-Hill, 2012; Cocuron et al., 2019; Carey et al., 2020; Tsogtbaatar et al., 2020). CCE results from the sum of all catabolic and anabolic metabolic processes which varies in developing embryos from different oilseeds, classified as “green” or “non-green”, depending on the presence or absence of chlorophyll during seed filling (Goffman et al., 2005; Allen et al., 2009; Lonien and Schwender, 2009; Carey et al., 2020; Tsogtbaatar et al., 2020). However, to decipher the biochemical pathways underlying the CCE in each species, a more quantitative analysis of the carbon fluxes through the central metabolism—which conducts the vast majority of biochemical carbon transformation—is needed. Steady state metabolic flux analysis (MFA) has been useful to gain a quantitative assessment of the carbon flux through central metabolism based on ^13^C-labeling, which may guide genetic engineering (Libourel & Shachar-Hill, 2008; Lee et al., 2011; Chen & Shachar-Hill, 2012; Kim et al., 2012; Kruger et al., 2012; O’Grady et al., 2012; Shachar-Hill, 2013). In parallel, several strategies have been employed to genetically manipulate FA composition and plant lipid metabolism in order to increase the FA content in oilseeds. To this end, the “push, pull, package, and protect” strategy has been implemented at various degrees; it consists of manipulating the expression of genes to boost the synthesis of FAs (“push”), or increase TG assembly reactions (“pull”), or improve the storage of FAs into lipid droplets (LDs) (“package”), or prevent the degradation of stored lipids (“protect”), or any combination of these.

This review focuses on FAS in seeds, highlighting the limitation and challenges involved in performing earlier experiments with isolated plastids and the relevance of ^13^C-MFA to decipher the pathways that contribute to oil biosynthesis, discussing how photochemical activity and unconventional pathways may contribute to higher efficiency of carbon conversion, assessing key genes and regulators that have been recently targeted to enhance seed oil content, and proposing alternative targets/strategies to achieve desirable oil content and quality.

## Fatty acid synthesis and elongation in seeds

**Figure 1:**
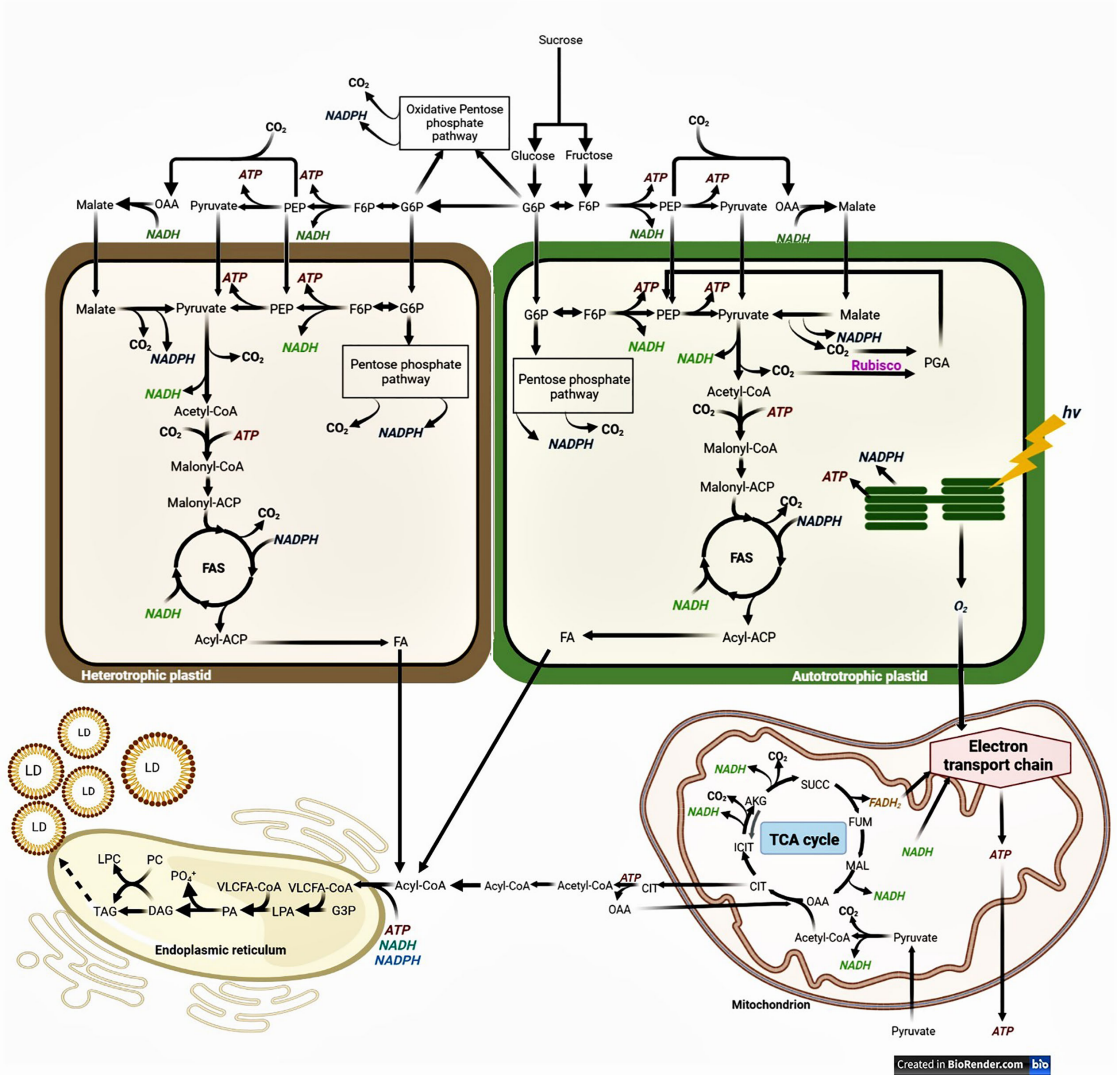
Simplified schematic biochemical pathway of fatty acid and TG synthesis in higher plants. Fatty acid synthesis (FAS) and lipid droplet (LD) formation in heterotrophic and in autotrophic embryos. ACP, acyl carrier protein; AKG, α-ketoglutarate; CIT, citrate; CoA, coenzyme A; DAG, diacylglycerol; FA, fatty acid; F6P, fructose 6-phosphate; FUM, fumarate; G6P, glucose 6-phosphate; *hv*, light; ICIT, isocitrate; LD, lipid droplet; LPA, lysophosphatidic acid; LPC, lysophosphatidylcholine; MAL, malate; OAA, oxaloacetate; PA, phosphatidic acid; PC, phosphatidylcholine; PGA, phosphoglycerate; PEP, phosphoenolpyruvate; PGA, phosphoglycerate; SUCC, succinate; TCA, tricarboxylic acid; TG, triacylglycerol; VLCFA, very-long-chain fatty acid.

The schematic mechanism of FAS and FA elongation for green and non-green embryos, and the possible sources of carbon precursors, energy, and reductants, are depicted in **Figure 1**. The disaccharide sucrose represents the major form in which photosynthetically assimilated carbon is transported into oil seeds. The hexose phosphates generated by the cleavage of sucrose can be metabolized through the glycolysis and the oxidative pentose phosphate pathway (OPPP) which can be found both in the cytosol and in the plastids. A major route for carbon going into FAS may involve the cytosolic glycolytic pathway until phosphoenolpyruvate (PEP) and pyruvate, which may be imported into the plastid and undergo decarboxylation to form acetyl-coenzyme A (acetyl-CoA) *via* the plastidic pyruvate dehydrogenase complex. Acetyl-CoA carboxylase (ACCase) is the first committed step for the FAS: it uses energy to carboxylate acetyl-CoA into malonyl-CoA which is transferred to the Acyl Carrier Protein (ACP) by the malonyl-CoA-ACP transacylase. Then, malonyl-ACP is condensed with acetyl-CoA by the 3-ketoacyl-ACP synthase III (KAS III), generating 3-ketobutyryl-ACP. The 3-ketoacyl-ACP reductase utilizes NADPH to reduce 3-ketobutyryl-ACP into 3-hydroxybutyryl-ACP which is dehydrated by 3-hydroxyacyl-ACP dehydratase to form trans-Δ2-butenoyl-ACP. The reduction of the double bond uses NAD(P)H to convert trans-Δ2-butenoyl-ACP into butyryl-ACP which in then condensed with malonyl-CoA by the 3-ketoacyl-ACP synthase I (KAS I) to generate 3-ketoacyl-ACP. This cycle is repeated to elongate saturated FA chains till 16:0-ACP, and then, the 3-ketoacyl-ACP synthase II (KAS II) performs the last elongation step to synthesize 18:0-ACP. The stearoyl-ACP Δ9-desaturase desaturates 18:0-ACP into 18:1-ACP. Plastidic *de novo* FAS ends when the FA thioesterase removes the ACP group from acyl backbones.

Beyond the complex network defined by interconnected OPPP and glycolytic pathways, additional metabolic routes may supply precursors for *de novo* FAS in the plastid **(** **Figure 1** **)**. During the conversion of pyruvate into acetyl-CoA, there is a concomitant loss of the carboxylic group of the pyruvate (released as CO_2_) which has a significant impact on the CCE of developing embryos. In autotrophic plastids of green embryos, ribulose-1,5-bisphophate carboxylase/oxygenase (Rubisco) is able to re-fix the released CO_2_ apart from the Calvin cycle. This “Rubisco shunt” involves the conversion of hexose-phosphates and triose-phosphates to ribulose-1,5-bisphosphate (RuBP) by the non-oxidative reactions of the OPPP, and the subsequent fixation of CO_2_ onto RuBP by Rubisco and cleavage in two 3-phosphoglycerate (PGA) (Schwender et al., 2004). PGA can be further metabolized to pyruvate and then to FAs *via* acetyl-CoA. This metabolic route offsets the loss of CO_2_ at reactions, such as the OPPP and pyruvate dehydrogenase, thus increasing the CCE in developing embryos. Another route for the synthesis of plastidial pyruvate (and then acetyl-CoA) is through decarboxylation of imported malate by the plastidial NADP-dependent malic enzyme (pNADP-ME) (Smith et al., 1992; Shearer et al., 2004). Supply of malate relies on either the translocation of mitochondrial malate or the carboxylation of PEP into oxaloacetate (OAA) *via* the cytosolic phosphoenolpyruvate carboxylase, followed by the conversion of OAA into malate catalyzed by NAD-dependent malate dehydrogenase (King et al., 1998) which happens in both green and non-green seeds. The production of acetyl-CoA from imported malate requires the successive action of the pNADP-ME and the plastidic pyruvate dehydrogenase, which presents the disadvantage of generating 2 molecules of CO_2_ for each 4-carbon malate molecule **(** **Figure 1** **)**.

FAS also requires stoichiometric amounts of ATP, NADPH, and NADH for each sequential addition of an acetyl unit to the growing chain of the fatty acid **(** **Figure 1** **)**. ATP is required for the carboxylation of acetyl-CoA to malonyl-CoA by ACCase, whereas the two reductases of the FAS complex require NADPH and NADH, respectively. Plastids of oilseeds must either take up ATP produced by oxidative phosphorylation in the mitochondria or generate it internally. ATP can be produced in the cytosol *via* the glycolysis and through the mitochondrial oxidative phosphorylation, and then be imported into the plastids *via* a nucleotide transporter. A potential source for ATP and reductant within the plastids is generated by the oxidation of sugar phosphates *via* glycolytic enzymes: glyceraldehyde 3-phosphate dehydrogenase generates NADH whereas phosphoglycerate kinase and pyruvate kinase produce ATP. Likewise, the subsequent conversion of pyruvate into acetyl-CoA by the pyruvate dehydrogenase is accompanied by the production of NADH. The intraplastidial conversion of malate to pyruvate by pNADP-ME constitutes another potential source of NADPH. Finally, the oxidation of sugar phosphates by the plastidial OPPP may also contribute to the production of NADPH. In the case of green oilseeds, light reactions in the thylakoids produce ATP and NADPH that can be used for *de novo* FAS **(** **Figure 1** **)**. The balance between cyclic and non-cyclic photophosphorylation may also adjust respective ATP and NADPH productions to metabolic requirements in autotrophic plastids (Allen et al., 2009).

Following FAS, acyl groups in FA are hydrolyzed by acyl-ACP thioesterases, releasing free acyl chains. These free acyl chains are then activated to CoA esters on the outer membrane of the plastid by long-chain acyl-CoA synthetases prior to their export toward the ER for their elongation into very-long chain fatty acids (VLCFA). Carbon for FA elongation comes from the cleavage of cytosolic citrate into OAA and acetyl-CoA by citrate lyase, reaction that requires ATP **(** **Figure 1** **)**. This cytosolic acetyl-CoA is then used by acetyl-CoA carboxylase to synthesize malonyl-CoA, the carbon donor for FA elongation. The elongation of acyl-CoA in the ER involves four sequential enzymatic steps catalyzed by the fatty acid elongase complex: i) the ketoacyl-CoA synthase (KCS) requires energy under the form of ATP to condenses malonyl-CoA with the elongating acyl-CoA; ii) the 3-ketoacyl-CoA reductase (KCR) uses (NAD/PH) to reduce 3-ketoacyl-CoA; iii) the 3-hydroxy acyl-CoA dehydrate (HCD) catalyzes the dehydration of 3-hydroxyacyl-CoA; and iv) the enoyl-CoA reductase (ECR) uses NAD/PH to reduce enoyl-CoA. Similar to FAS, FA elongation requires energy and reductant. ATP may be produced by oxidative phosphorylation in the mitochondria and cytosolic glycolysis which may also provide NADH. The cytosolic pentose-phosphate pathway may contribute to NADPH production in developing embryos with the glucose-6-phosphate and 6-phosphogluconate dehydrogenases (**Figure 1**).

Finally, the assembly of TGs involves the sequentially esterification of acyl-CoAs to glycerol 3-phosphate (G3P) backbone by membrane-bound acyltransferases **(** **Figure 1** **)**. The first acylation yields lysophosphatidic acid (LPA), which in turn is acylated to produce the central metabolite phosphatidic acid (PA). PA is then converted to diacylglycerol (DAG) by the action of PA phosphatase (PAP). In the Kennedy pathway, a third FA is transferred to the vacant position of DAG by diacylglycerol acyltransferase (DGAT), the only enzymatic reaction of the pathway exclusively committed to TG biosynthesis (Cao and Huang, 1986; Cao and Huang, 1987; Li-Beisson et al., 2013). The acyl chains from phosphatidylcholine (PC) may also become available for TG synthesis through transfer of an acyl from PC to DAG, which is catalyzed by phospholipid:diacylglycerol acyltransferase (PDAT). Once TG assembly is achieved in the ER, TGs are accumulated between the two layers of phospholipids, resulting in the formation of structures called oil bodies or lipid droplets (LD). These spherical organelles comprise a matrix of TGs surrounded by a phospholipid monolayer where the aliphatic chains are oriented to the TG lumen and the phosphate groups toward the cytosol (Yatsu and Jacks, 1972; Chapman and Ohlrogge, 2012).

## Identification of sources of carbon precursors and reductants for FAS using isolated plastids

Besides ATP which can be produced by oxidative phosphorylation and can be translocated from one compartment to the other, the carbon precursor (acetyl-CoA) and reductant do not cross biological membranes, and have therefore to be synthesized in the plastids for FAS and/or the cytosol for FA elongation. As described above (**Figure 1**), several biochemical steps may lead to the production of acetyl-CoA and NAD(P)H, and their relative importance varies from one species to another. Determining the nature of carbon precursors and reducing power for FAS is crucial to understand and enhance oil synthesis in developing seeds, and also to identify potential bottlenecks.

Early experiments were performed by incubating isolated plastids with radiolabeled substrates to investigate which carbon precursors were stimulating the rate of *de novo* FAS, and which pathway were producing reductant. Feeding isolated plastids from *Brassica napus* with ^14^C-labeled substrates showed that pyruvate, glucose 6-phosphate (G6P), dihydroxyacetone phosphate (DHAP), malate, acetate, and phosphoenolpyruvate (PEP) were utilized as precursors for FAS (Kang and Rawsthorne, 1994; Kubis et al., 2004). Particularly, the utilization of G6P through the OPPP also provided reductant for FAS (Eastmond and Rawsthorne, 2000; Pleite et al., 2005). However, incubation of *B. napus* isolated plastids with ^14^C-PEP revealed that it did not only provide carbons, but also supplied additional ATP for FAS (Kubis et al., 2004). In contrast to *B. napus*, feeding sunflower isolated plastids with ^14^C-G6P indicated that there was no incorporation of labeling in FAs (Pleite et al., 2005). However, pyruvate utilization in combination with G6P dramatically increased FAS. Due to an insufficient NADPH pool, pyruvate feeding alone was not sufficient to enhance the rate of FAS. Moreover, malate has been demonstrated to be one of the most efficient precursors for FAS. Indeed, in isolated plastids from castor and sunflower embryos, supply of malate stimulated the rates of FAS (Smith et al., 1992; Pleite et al., 2005). In comparison to pyruvate and acetate, malate significantly increased the rate of FAS due to generation of additional NADPH *via* pNADP-ME (Pleite et al., 2005). However, supply of G6P with malate did not significantly alter the rate of FAS in isolated plastids.

Although experiments conducted with isolated plastids were key to identify the carbon precursors and reductant, these results did not completely reflect what happens *in vivo*, in the entire developing embryo. Initial experiments *in vivo* were carried out by incubating embryos with radioisotopes. Labeling studies performed with [U-^14^C_4_]-malate in sunflowers embryos in culture showed that malate could provide a source of carbon for the FAS (Alonso et al., 2007), corroborating the previous results on isolated plastids mentioned above (Pleite et al., 2005). However, malate is not a physiological substrate provided from the sunflower plant to the developing embryos. Using culture conditions that mimic the feeding and the development of the sunflower embryos *in planta*, isotopic steady state labeling experiments demonstrated that the flow of malate towards FAS was minor; the major source of carbon (91-95%) contributing to FAS was from triose phosphates (Alonso et al., 2007). Due to discrepancies between results on isolated plastids and whole embryos in culture, MFA has been the method of choice to study the metabolic pathways involved in FAS under conditions that are physiologically relevant.

## Metabolic flux analysis to study fatty acid synthesis in developing embryos

### Accessing C partitioning *in vivo* using ^13^C-MFA

The goal of MFA is to quantify all the *in vivo* metabolic fluxes in a given organ or cell, here developing embryos, which results in a metabolic flux map. The determination of intermediary carbon fluxes requires ^13^C-labeling. Therefore, establishing culture conditions that mimic the development of embryos *in planta* is decisive to build carbon flux maps. For instance, it has been found for multiple species of the Brassicaceae family that developing embryos readily grow in liquid cultures (Schwender and Ohlrogge, 2002; Lonien and Schwender, 2009; Chen and Shachar-Hill, 2012; Tsogtbaatar et al., 2020). Substrates, furnished by the mother plant, are unloaded in the endosperm liquid and taken up by the embryo. Therefore, the best way to design a liquid growth medium that mimics the *in planta* liquid environment is to analyze the constituents of the endosperm or the vascular tissue (Alonso et al., 2007; Alonso et al., 2010; Cocuron et al., 2019; Tsogtbaatar et al., 2020). Substrate composition, total osmotic pressure of the medium, and light intensity are important factors influencing plant tissue development (Goffman et al., 2005; Allen et al., 2007; Alonso et al., 2010), and hence have to be optimized. To meet requirements for ^13^C-MFA, it is important to maintain homeostasis and metabolic steady state in culture embryos. Therefore, photoperiod is not commonly applied to embryos in culture for ^13^C-MFA studies. Ideal culture conditions for plant embryos are validated when the dry weight gain and the biomass composition of embryos grown in culture are not significantly different from the ones grown *in planta* (Alonso et al., 2007; Alonso et al., 2010; Alonso et al., 2011; Cocuron et al., 2019; Tsogtbaatar et al., 2020). Parallel labeling experiments, using different ^13^C-substrates, are usually conducted to have a better coverage of the metabolic network (Schwender et al., 2006; Alonso et al., 2007; Alonso et al., 2010; Antoniewicz, 2015; Crown et al., 2016; Antoniewicz, 2018; Acket et al., 2019; Tsogtbaatar et al., 2020). Labeled embryos are harvested after they have reached an isotopic steady-state, meaning that the labeling in intermediary metabolites and products have reached a constant pattern. The resultant labeling in a range of metabolites is then determined using nuclear magnetic resonance and/or mass spectrometry (MS) (Ratcliffe and Shachar-Hill, 2006; Dieuaide-Noubhani and Alonso, 2014). Several sensitive gas chromatography-mass spectrometry (GC-MS) and liquid chromatography tandem mass spectrometry (LC-MS/MS) methods have been recently developed to follow the labeling directly in key metabolic intermediaries such as sugars, phosphorylated compounds, free amino acids, and organic acids (Alonso et al., 2010; Koubaa et al., 2013; Cocuron and Alonso, 2014; Cocuron et al., 2017; Acket et al., 2019; Cocuron et al., 2020). Compartmentalization in plant cells is usually considered by following different labeling patterns of a few metabolites and hydrolyzed macromolecules whose biosynthesis occur in one compartment (Allen et al., 2007; Allen et al., 2012; Dieuaide-Noubhani and Alonso, 2014; Cocuron et al., 2020). The complete labeling information is entered into a mathematical model describing the metabolic network. This mathematical model includes equations expressing metabolic and isotopic steady states. Finally, the fluxes through the network that correspond to the observed label distribution are calculated using mathematical algorithms that can be computed using available software (Wiechert and De Graaf, 1997; Wiechert and De Graaf, 1997; Möllney et al., 1999; Wiechert et al., 1999; Wiechert et al., 2001). ^13^C-based MFA has been successfully applied to plant systems to characterize the *in vivo* carbon fluxes in important metabolic pathways during FAS in developing embryos (Goffman et al., 2005; Alonso et al., 2007; Allen et al., 2009; Lonien and Schwender, 2009; Alonso et al., 2010; Cocuron et al., 2019; Carey et al., 2020; Tsogtbaatar et al., 2020).

### Determination of carbon conversion efficiency in developing embryos

Determining the efficiency of the developing embryo in converting carbon sources into oil and other biomass components (e.g. proteins, and carbohydrates) known as carbon conversion efficiency (CCE), is important to evaluate the potential for improving seed oil quantity (Goffman et al., 2005; Alonso et al., 2007; Allen et al., 2009; Lonien and Schwender, 2009; Chen and Shachar-Hill, 2012; Cocuron et al., 2019; Carey et al., 2020; Tsogtbaatar et al., 2020). CCE results from the sum of all catabolic and anabolic metabolic processes, which varies in developing embryos from different oilseeds (Goffman et al., 2005; Allen et al., 2009; Lonien and Schwender, 2009; Carey et al., 2020; Tsogtbaatar et al., 2020). To assess the CCE, embryos are grown for several days culture media and conditions that mimic their development in plant, as explained in the above section. There are two options to determine the percentage of carbon uptaken that was stored into biomass components. The first option uses ^14^C-labeled substrates in sealed flasks, and quantifies the radiolabeling released as ^14^CO_2_, and incorporated as ^14^C-oil, ^14^C-proteins, and ^14^C-carbohydrates (Goffman et al., 2005; Alonso et al., 2007; Allen et al., 2009; Alonso et al., 2010; Alonso et al., 2011; Carey et al., 2020). The second option is the quantify the substrate depletion from the media and the biomass stored during the incubation period (Cocuron et al., 2019; Tsogtbaatar et al., 2020). **Table 1** summarizes the biomass composition and the CCE of different embryos from green and non-green oilseeds that have been studied and published so far. Under physiological conditions, the biomass composition of the developing embryos in culture differs among species. Oil content varied from 18% (w/w) for *G. max* to 56% for *B. napus*, proteins from 6% for *Z. mays* LH59 to 40% for *G. max*, and carbohydrates from 43% for *G. max* to 60% for *Z. mays* LH59. Under physiological conditions, *T. arvense* embryos were found to be the most efficient in converting carbon substrates into biomass (93%) while *C. sativa* were the least (32%). It is important to note that these differences in CCE reflect the amount of carbon loss as CO_2_ during the synthesis of biomass components: the lower the CCE, the higher is the loss of carbon as CO_2_, which has implications for metabolic engineering. For instance, improving oil content in *C. sativa* may be achieved by reducing the pathways producing CO_2_. However, this strategy would not work in *T. arvense* which is already extremely efficient. Instead, for *T. arvense*, one would have to redirect carbon from another biomass component to increase FAS.

**Table 1:** Biomass composition and carbon conversion efficiency.

Species	*Biomass composition in embryo (% w/w)*			CCE (%)	References
	Oil	Protein	Carbohydrates		
*Zea mays* LH59 (low oil line)*	34	6	60	57-71	(Alonso et al., 2010)
*Zea mays* ALEX (high oil line)*	48	13	39	61-64	(Cocuron et al., 2019)
Helianthus annuus*	38	18	44	50	(Alonso et al., 2007)
*Glycine max*, 35 µE*	18	39	43	83	(Allen et al., 2009)
*Brassica napus*, dark	45			60	(Goffman et al., 2005)
*Brassica napus*, 50 µE*	56			86	(Goffman et al., 2005)
*Brassica napus*, 150 µE	58			95	(Goffman et al., 2005)
*Thlaspi arvense*, 20 µE*	31	28	41	93	(Tsogtbaatar et al., 2020)
*Camelina sativa*, dark	29	23	48	21	(Carey et al., 2020)
*Camelina sativa*, 10 µE*	35	27	38	32	(Carey et al., 2020)
*Camelina sativa*, 50 µE	34	20	46	42	(Carey et al., 2020)
*Arabidopsis thaliana* (Col), 50 µE	41	24	13	80	(Lonien and Schwender, 2009)

Comparison of the biomass composition and carbon conversion efficiency (CCE) in embryos from crop and model species grown under cultured conditions. Embryos from species highlighted in green are green embryos that may be photosynthetically active; the * denotes physiological conditions.

Except *C. sativa*, embryos from green seeds had higher CCEs than those seeds that cannot use light (**Table 1**). The importance of light was further demonstrated by incubating developing embryos at different intensities. In general, higher light intensity significantly increased the CCE. This finding suggests that light has strong effects on the metabolism of developing green embryos—probably due to the additional production of NADPH and ATP *via* photosynthesis–resulting in faster growth and storage product accumulation, which improved the CCE (Goffman et al., 2005; Alonso et al., 2007; Allen et al., 2009; Lonien and Schwender, 2009; Chen and Shachar-Hill, 2012; Cocuron et al., 2019; Carey et al., 2020; Tsogtbaatar et al., 2020). Indeed, studies on these developing green embryos showed that all the major photosynthetic complexes (PSII, PSI and their antenna complexes, cytochrome b6f complex, and ATP synthase) are present at a necessary stoichiometric ratio, suggesting a high photochemical activity despite being partially blocked from light by the pod and seed coat (Asokanthan et al., 1997; Ruuska et al., 2004; Puthur et al., 2013; Allorent et al., 2015). The light reactions occurring in these green embryos have been associated with the rapid synthesis of ATP and NADPH needed for energetically-expensive FAS. It has also been reported that embryo photosynthesis contributes to a significant amount of oxygen, which fuels energy-generating biochemical pathways, including mitochondrial respiration (Ruuska et al., 2004; Borisjuk et al., 2005; Rolletschek et al., 2005; Tschiersch et al., 2011; Galili et al., 2014).

Differences in the flow of carbon through central metabolic pathways are responsible for the differences in biomass composition and CCE measured in developing embryos from various oilseed species (**Table 1**). ^13^C-MFA has been the method of choice to measure *in vivo* rates of carbon flow, quantifying the metabolic pathways that are active during FAS.

### MFA to identity carbon sources and reductants for FAS in developing embryos

^13^C-MFA was applied to developing embryos from various oilseed species to determine the sources of carbon and reductant for FAS, and to unravel the occurrence of non-conventional pathways that improved the CCE (**Table 2**) (Schwender et al., 2004; Schwender et al., 2006; Alonso et al., 2007; Allen et al., 2009; Lonien and Schwender, 2009; Alonso et al., 2010; Hay et al., 2014; Cocuron et al., 2019; Acket et al., 2020; Carey et al., 2020; Tsogtbaatar et al., 2020). Glycolysis is a major source of pyruvate for *de novo* FAS (**Figure 1**). ^13^C-labeling and metabolic flux analysis performed in non-photosynthetic and photosynthetic embryos revealed the contribution of the plastidic NADP-dependent malic enzyme (pNADP-ME) for the production of plastidic pyruvate (pPYR) in *Z. mays*, *H. annuus, G. max, T. arvense*, and *C. sativa* (**Table 2**). The pNADP-ME provided up to 54% of pPYR in the high oil ALEX maize line (Cocuron et al., 2019). In plastids, pNADP-ME catalyzes the conversion of malate to pPYR with the production of CO_2_ and NADPH (**Figure 1**). In addition, pPDH further catalyzes the decarboxylation of pPYR to acetyl-CoA with the generation of CO_2_ and NADH. Although the overall equimolar conversion of malate into plastidic acetyl-CoA results in the production of valuable reductant necessary for FAS (NADH and NADPH), it leads, in return, to the loss of two carbons as CO_2_, which may affect the overall CCE. Interestingly, ^13^C-labeling in developing *B. napus* embryos demonstrated for the first time that Rubisco was fixing this plastidic ^13^CO_2_ to produce phosphoglycerate (pPGA) through an unconventional “Rubisco shunt” (**Table 2**) (Schwender et al., 2004). The fixation of pCO_2_ by Rubisco contributes to additional sources of carbon for FAS: it compensates for the decarboxylation steps, and channels more substrates into FAS, improving the CCE (Eastmond et al., 1996; Ruuska et al., 2004; Schwender et al., 2004). Indeed, studies in developing *B. napus* embryos showed a CCE of 86% under physiological conditions due to CO_2_ refixation by Rubisco into pPGA (**Tables 1**, **2**) (Schwender et al., 2004). ^13^C-MFA demonstrated that up to 64% of pPGA was produced by Rubisco, contributing to the synthesis of acetyl-CoA for *de novo* FAS, and reducing by 40% the carbon lost as CO_2_ (**Table 2**) (Schwender et al., 2004). Similar processes have been described in developing embryos of *G. max* and *T. arvense* where Rubisco was found to contribute to 14% and 25% of the pPGA, respectively (**Table 2**) (Allen et al., 2009; Tsogtbaatar et al., 2020).

**Table 2:** Comparison of the use of non-conventional pathways in developing embryos.

Species	Contribution of pNADP-ME to pPYR	Rubisco contribution to pPGA	Reversibility of IDH	Contribution to NADPH for FAS	References
*Zea mays*	30-54%	0%	No	74-76% OPPP 30-55% pNADP-ME	(Alonso et al., 2010; Cocuron et al., 2019)
*Helianthus annuus*	7%	0%	No	212% OPPP 6% pNADP-ME	(Alonso et al., 2007)
*Glycine max*	<20%	14%	Yes	<24% OPPP <29% pNADP-ME	(Allen et al., 2009)
*Brassica napus*	<1%	36-64%	Yes	25% OPPP <1% pNADP-ME	(Schwender et al., 2004; Schwender et al., 2006; Hay et al., 2014)
*Thlaspi arvense*	20%	25%	Yes	n.d.	(Tsogtbaatar et al., 2020)
*Camelina sativa*	9%	0%	Yes	6,079% OPPP 15% pNADP-ME	(Carey et al., 2020)
*Linum usitatissinum*	<1%	0%	Yes	186% OPPP <1% pNADP-ME	(Acket et al., 2019)

Published data from ^13^C-labeling and metabolic flux analysis obtained from developing embryos cultured under physiological conditions were used to determine the contribution of the plastidic NADP-dependent malic enzyme (pNADP-ME) to the production of plastidic pyruvate (pPYR); the contribution of Rubisco to plastidic phosphoglycerate (pPGA); the reversibility of the isocitrate dehydrogenase (IDH); and the contribution of the oxidative pentose-phosphate pathway (OPPP) and pNADP-ME to the production of NADPH necessary for fatty acid synthesis (FAS). Embryos from species highlighted in green are green embryos that may be photosynthetically active.

In addition to the role of Rubisco, ^13^C-labeling and MFA also revealed the non-canonical function of isocitrate dehydrogenase (IDH) in photosynthetic embryos **(** **Table 2** **)**. This reaction, assumed to be thermodynamically irreversible, was reported to catalyze the carboxylation of α-ketoglutarate into isocitrate *in vivo* in developing *B. napus* embryos (Schwender et al., 2006). It is important to note that the CO_2_ fixation by IDH may also improve the CCE. This phenomenon was explained by a high demand in citrate for FA elongation in *B. napus* (**Figure 1**), and a high concentration of CO_2_ (40 mM) available in developing oilseeds (Goffman et al., 2004). Similarly, reversibility of IDH was measured in developing green embryos from *G. max*, even when the concentration of CO_2_ was lower (Allen et al., 2009), *T. arvense* (Tsogtbaatar et al., 2020), *C. sativa* (Schwender et al., 2006), and *L. usitatissinum* (Acket et al., 2019), but absent in heterotrophic embryos (**Table 2**). Interestingly, developing embryos that have an active Rubisco and reversible IDH were found to be the more efficient in converting their substrates into biomass (**Tables 1**, **2**).

Besides carbon, FAS requires reductant that must be synthesized in the plastid. For each mole of acetyl-CoA produced, the plastidic pyruvate dehydrogenase complex generated one mole of NADH, which can be directly used for FAS (**Figure 1**). The plastidic production of NADPH necessary for *de novo* FAS may be ensured by the OPPP and/or pNADP-ME, and light reactions in the case of photosynthetic embryos (**Figure 1**). Knowing that the OPPP and the pNADP-ME generate CO_2_, their operation may affect negatively the CCE (**Figure 1**). ^13^C-labeling and MFA were used to measure the relative contribution of the OPPP and pNADP-ME to the production of NADPH in developing embryos from different species **(** **Table 2** **)**. In general, the OPPP supported a higher production of NADPH than the pNADP-ME. For *G. max* and *B. napus*, these two combined pathways did not provide sufficient NADPH to support FAS in developing embryos. In those species, the remainder of NADPH may be supplied by the light reactions of photosynthesis and/or catabolism (Goffman et al., 2005; Schwender et al., 2006; Allen et al., 2009). For *Z. mays*, the production of NADPH by the OPPP and pNAPD-ME was just enough to support FAS in developing embryos (**Table 2**). More specifically, the pNAPD-ME was found to work at maximal capacity *in vivo*; it was identified as the limiting step in the provision of pPYR and NADPH for FAS in maize (Alonso et al., 2010; Cocuron et al., 2019). The embryos from the other species, *H. annuus*, *C. sativa*, and *L. usitatissinum*, were producing NADPH *via* the OPPP and pNADP-ME in excess of the requirements for FAS (**Table 2**), concomitantly generating CO_2_, which resulted in the lowest CCE for these species (**Table 1**).

Overall, the aforementioned studies have demonstrated that MFA is a valuable tool to quantify the carbon partitioning *in vivo*, and identify the sources of carbon skeletons and reductants necessary for FAS in the developing green and non-green embryos. Information gathered from MFA, combined with transcriptomics and metabolomics, will give insights on the genes that control oil synthesis and create novel approaches for the genetic engineering of oilseed crops. The following sections review some of the target genes and the common genetic engineering strategies for enhancing oil content and quality in seeds.

## Metabolic engineering strategies to improve seed oil content and composition

**Figure 2:**
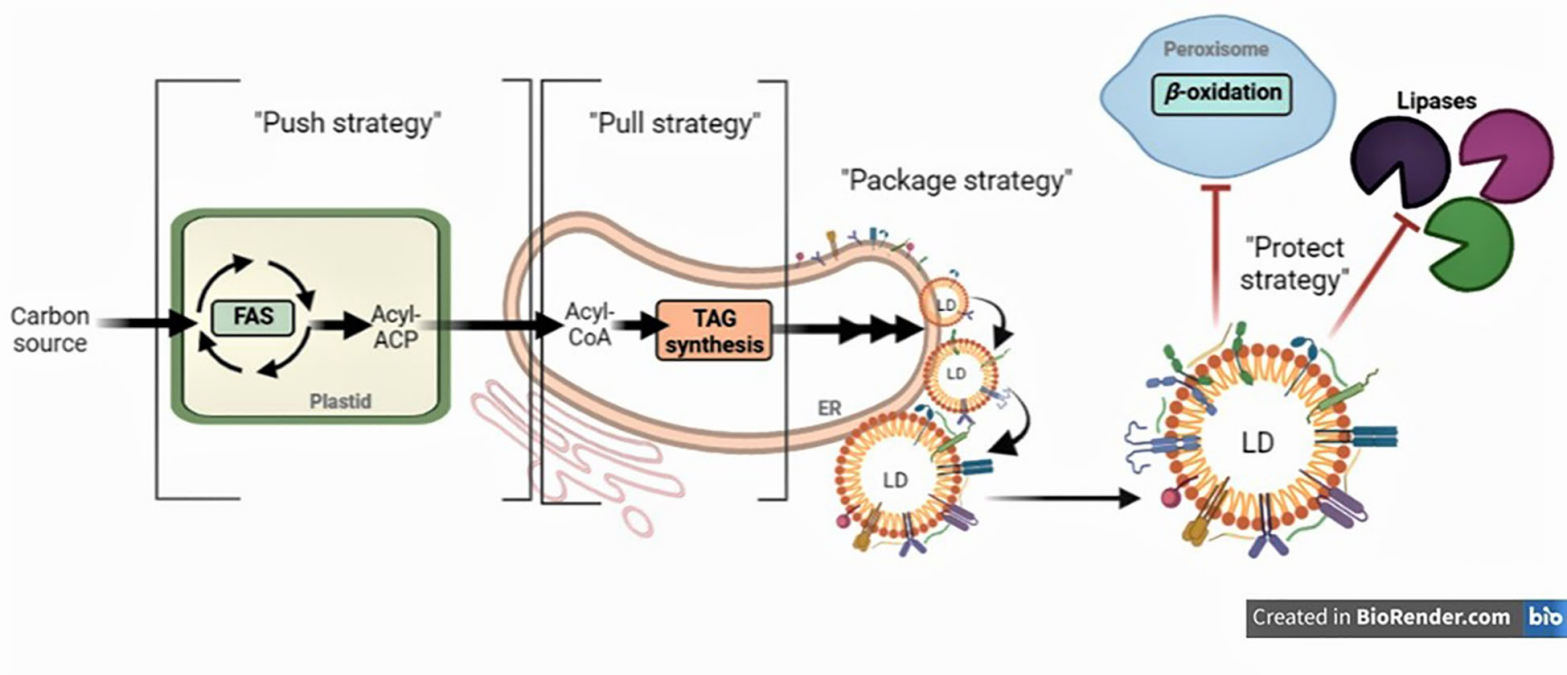
Improving oil quality in seeds by an integrated metabolic engineering approach. Broadly, strategies aim to (1) increase the carbon flux into *de novo* fatty acid synthesis (FAS) (“Push strategy”), (2) ensure efficient assembly of nascent acyl chains into triacylglycerol (TG) (“Pull strategy”), (3) facilitate lipid droplet (LD) biogenesis and maximize droplet stability by proper coating (“Package strategy”), and (4) minimize TG turnover (“Protect strategy”).

To improve the yield and FA composition in oilseed crops, it is critical to understand how assimilates are partitioned in favor of storage lipids. It is also of great importance to elucidate the mechanisms of TG biosynthesis in different oilseeds to further optimize their FA composition since it is a key determinant of oilseed nutritional value and industrial applications. Several strategies have been employed to genetically manipulate FA composition and plant lipid metabolism in order to increase the FA content oilseeds. To this end, the “push, pull, package, and protect” strategy has been implemented at various degrees; it consists of manipulating the expression of genes to boost the synthesis of FAs (“push”), or increase TG assembly reactions (“pull”), or improve the storage of FAs into lipid droplets (LDs) (“package”), or prevent the degradation of stored lipids (“protect”), or any combination of these (**Figure 2**) (Vanhercke et al., 2019). The production of novel/unusual FAs presents additional challenges because they may disrupt membrane stability. Therefore, seeds must incorporate and store novel/unusual FAs into TG. **Tables 3**–**7** review the genes that were up- or down-regulated in several species, and when available, their effect in total seed oil content and FA composition.

**Table 3:** List of genes encoding TFs manipulated to enhance oil yield and/or change the FA composition in seeds.

Gene	Gene source	Target species	Strategy	Effect in seed oil content	Effect in fatty Acid composition	References
*ABI3*	*A. thaliana*	*A. thaliana*	Down-regulation	↘ seed oil content by up to 30%	↘ C20:1	(Yang et al., 2022)
			Down-regulation	↘ seed oil content by up to 42%	↘ C16:0, C16:1, C18:0, C18:1, C18:2, C18:3, C20:0, C20:1, C22:0, C22:1	(Roscoe et al., 2015)
*Dof1*	*G. hirsutum*	*G. hirsutum*	Over-expression	↗ seed oil content by 16%		(Su et al., 2017)
*DREBL*	*G. max*	*A. thaliana*	Over-expression	↗ seed oil content by 10%	↗ C18:1, C18:2, C18:3, C20:1	(Zhang et al., 2016)
*FUS3*	*B. napus*	*B. napus*	Down-regulation	↘ seed oil content by up to 12%	↗ C18:2	(Elahi et al, 2015)
	*A. thaliana*	*A. thaliana*	Down-regulation	↘ seed oil content by up to 67%	↘ C16:0, C16:1, C18:0, C18:1, C18:2, C18:3, C20:0, C20:1, C22:0, C22:1	(Roscoe et al., 2015)
*GLABRA2 (GL2)*	*A. thaliana*	*A. thaliana*	Down-regulation	↗ seed oil content by 7%		(Shi et al., 2012)
*GRF2*	*B. napus*	*A. thaliana*	Over-expression	↗ seed oil content by 48%		(Liu et al., 2012)
*LEC1*	*Z. mays*	*Z. mays*	Over-expression	↗ seed oil content by 48%		(Shen et al., 2010)
	*B. napus*	*B. napus*	Over-expression	↗ seed oil content by 2%	↗ C16:0, C18:0, C18:2, C18:3; ↘ C18:1	(Tan et al., 2011)
	*Z. mays*	*A. thaliana*	Over-expression	↗ seed oil by 20%		(Zhu et al., 2018)
		*C. sativa*	Over-expression	↗ seed oil by more than 26%		(Zhu et al., 2018)
	*B. napus*	*B. napus*	Over-expression	↘ seed oil content by up to 16%	↗ C18:1, C18:3; ↘ C16:0	(Elahi et al., 2016)
			Down-regulation	↘ seed oil content by up to 12%	no significant difference	(Elahi et al., 2016)
	*A. thaliana*	*A. hypogea*	Over-expression	↗ seed oil by up to 16%	↗ C18:1, C18:2, C18:3, C20:1; ↘ C16:0, C18:0	(Tang et al., 2018)
*LEC2*	*G. max*	*A. thaliana*	Over-expression	↗ seed oil content by 34%	↗ C18:0, C18:1; ↘ C16:0, C18:2, C20:1	(Manan et al., 2017)
*L1L*	*B. napus*	*B. napus*	Over-expression	↗ seed oil content by 16%	↗ C16:0, C18:0, C18:2, C18:3; ↘ C18:1	(Tan et al., 2011)
*MED15*	*A. thaliana*	*A. thaliana*	Over-expression	↗ seed oil content by up to 15%	↘ C20:1	(Kim et al., 2016)
			Down-regulation	↘ seed oil content by up to 30%	↘ C18:2; ↗ C18:3, C20:0	(Kim et al., 2016)
*MIF1*	*A. thaliana*	*A. thaliana*	Over-expression	↗ seed oil content by 13%	↗ C18:1, C20:0, C20:1; ↘ C18:3, C20:2, C20:3	(Cheng et al., 2021)
			Down-regulation	no significant difference	no significant difference	(Cheng et al., 2021)
*MYB1*	*J. curcas*	*A. thaliana*	Over-expression	↗ seed oil content by up to 28%	↗ C18:3, C20:1; ↘ C18:1	(Khan et al., 2019)
*MYB115* or *MYB118*	*A. thaliana*	*A. thaliana*	Down-regulation		↘ Total omega-7 fatty acids	(Troncoso-Ponce et al., 2016)
*MYB89*	*A. thaliana*	*A. thaliana*	Down-regulation	↗ seed oil content by up to 30%	↗ C16:0, C18:0, C18:1, C18:2; C18:3; C20:0; C20:1, C22:1	(Li et al., 2017)
*MYB96*	*C. sativa*	*C. sativa*	Over-expression	↗ seed oil content by up to 21%		(Kim et al., 2019)
			Down-regulation	↘ seed oil content by up to 15%	↘ C16:0, C18:0, C18:2, C18:3, C20:1, C20:2	(Lee et al., 2018)
*WRI1*	*A. thaliana*	*L. campestre*	Over-expression	↗ seed oil by up to 16% in T3 seeds	↘ C18:1, C18:2, C20:1, C22:1	(Ivarson et al., 2017)
	*B. napus*	*A. thaliana*	Over-expression	↗ seed oil by 10–40%		(Liu et al., 2010)
	*A. thaliana*	*C. sativa*	Over-expression	↗ seed oil by 8-31%	↗ C18:2, C18:3; ↘ C18:1, C20:1	(An and Suh, 2015)
	*Z. mays*	*Z. mays*	Over-expression	↗ seed oil by 9%	↗ C16:0, C18:0, C18:1, C18:3; ↘ C9:0, C10:0	(Pouvreau et al., 2011)
	*G. max*	*G. max*	Over-expression	↗ seed oil content by up to 13%	↗ C18:1, C18:2	(Chen et al., 2018)
	*C. nucifera*	*A. thaliana*	Over-expression	↗ seed oil content by up to 20%	↗ C16:0, C18:0, C18:1; ↘ C20:1	(Sun et al., 2017)
	*C. nucifera*	*O. sativa*	Over-expression	↗ seed oil content by up to 7%	↗ C16:0, C18:3; ↘ C18:1	(Sun et al., 2017)
	*Z. mays*	*Z. mays*	Over-expression	↗ seed oil by 46%	no significant difference	(Shen et al., 2010)
	*H. annuus*	*A. thaliana*	Over-expression	↗ seed oil by 40%		(Lim et al., 2022)
	*A. thaliana*	*A. thaliana*	Down-regulation	↘ seed oil content by up to 33%	↗ C18:3, C22:1; ↘ C18:1, C18:2, C20:1	(Baud et al., 2007)
*WRKY6*	*A. thaliana*	*A. thaliana*	Down-regulation	↗ seed oil content by 25-27%	↗ C18:3, C20:0; ↘ C18:2	(Song et al., 2020)

### Push strategy

This strategy aims to enhance the flux of carbon through FAS to produce additional acyl chains in the plastids for subsequent assembly into TGs in the ER (**Figure 2**). The “push” strategy is considered to be one of the most extensively used to manipulate oil content. Commonly, over-expression (or even down-regulation) of transcription factors (TFs) offers a great advantage of controlling a number of reactions simultaneously in the oil biosynthetic pathway (**Table 3**). The best examples of such TFs are LEAFY COTYLEDON1 (*LEC1*), LEC1-LIKE (*L1L*), *LEC2*, and WRINKLED1 (*WRI1*) (Focks and Benning, 1998; Lotan et al., 1998; Stone et al., 2001; Kwong et al., 2003; Cernac and Benning, 2004; Mu et al., 2008). Other TFs, shown to regulate the expression of genes involved in FAS, have been tested, including dehydration-responsive element-binding (*DREB*) (Zhang et al., 2016), Dof-type transcription factor 1 (*Dof1*) (Su et al., 2017), FUSCA3 (*FUS3*) (Elahi et al, 2015), growth-regulating factor 2-like (*GRF2*) (Liu et al., 2012), GLABRA2 (GL2) (Shi et al., 2012), and MYB interaction factor 1 (*MIF1*) (Cheng et al., 2021). Among all the TFs, *GRF2, LEC1, WRI1* were shown to be the most efficient in increasing seed oil content (40% to 48% higher compared to wild-type) (**Table 3**). In parallel, specific enzymes in the first committed steps of *de novo* FAS have also been targeted, such as acetyl-CoA carboxylase (ACCase) (Dong et al., 2002; Cui et al., 2017), and malonyl CoA-ACP malonyltransferase (MCAMT) (Jung et al., 2019) (**Table 4**). Finally, several studies demonstrated that overexpression of membrane-intrinsic proteins that mediate the export of FAs from plastids, such as FA export 1 (*FAX1*) (Li et al., 2015b; Tian et al., 2018; Li et al., 2020b; Cai et al., 2021), *FAX2* and *FAX4* (Li et al., 2015b; Li et al., 2016a; Li et al., 2020b), BILE ACID : SODIUM SYMPORTER FAMILY PROTEIN 2 (BASS2) (Lee et al., 2017), and fatty acyl-ACP thioesterases (FAT) (Bonaventure et al., 2003; Sinha et al., 2007; Parveez et al., 2010; Belide et al., 2012; Nguyen et al., 2015a; Ozseyhan et al., 2018; Ma et al., 2021; Liu et al., 2022) could also boost lipid accumulation in seeds (**Table 4**). Using the “push” strategy, the highest TG levels recorded in seed so far were achieved by over-expressing ACCase in maize (+65%**)** (Dong et al., 2002) (**Table 4**).

**Table 4:** List of “push” genes manipulated to enhance oil yield and/or change the FA composition in seeds.

Gene	Gene source	Target species	Strategy	Effect in seed oil content	Effect in fatty Acid composition	References
α-carboxyltransferase	*P. sativum*	*A. thaliana*	Overexpression	↗ seed oil content by up to 14%		(Wang et al., 2022)
		*C. sativa*	Overexpression	↗ seed oil content by up to 14%	↗ C18:1, C18:3; ↘ C16:0, C18:2	(Wang et al., 2022)
*ACCase*	*G. hirsutum*	*G. hirsutum*	Overexpression	↗ seed oil content by 17-22%		(Cui et al., 2017)
	*S. italica*	*Z. mays*	Overexpression	↗ seed oil content by 54-65%		(Dong et al., 2002)
*AAD*	*B. napus*	*B. napus*	Down-regulation	↘ seed oil content by up to 30%	↘ C18:1, C20:1	(Bryant et al., 2016)
*AAD +* *FAE1_hairpin +* *Fat5 +* *FatB_hairpin + KASII_hairpin*	synthetic *A. thaliana* *C. elegans* *C. sativa* *C. sativa*	*C. sativa*	Overexpression	no significant difference	↗ Total omega-7 fatty acids	(Nguyen et al., 2015)
		*G. max*	Overexpression	no significant difference	↗ Total omega-7 fatty acids	(Nguyen et al., 2015)
*AAD2* or *AAD3*	*A. thaliana*	*A. thaliana*	Overexpression		↗ C16:1 (omega-7), C18:1 (omega-7), C20:1 (omega-7)	(Ettaki et al., 2018)
			Down-regulation		↘ C16:1 (omega-7), C18:1 (omega-7), C20:1 (omega-7)	(Bryant et al., 2016)
*BASS2*	*A. thaliana*	*A. thaliana*	Overexpression	↗ seed oil content by 10-37%	no significant difference	(Lee et al., 2017)
	*B. napus*	*B. napus*	Overexpression	↗ seed oil content by 12%	↗ C18:0, C18:1; ↘ C16:0, C18:2	(Tang et al., 2022a)
			Down-regulation	↘ seed oil content by up to 11%	↗ C16:0, C18:0, C18:2; ↘ C18:1, C20:1	(Tang et al., 2022a)
*FATA*	*J. curcas*	*A. thaliana*	Overexpression	↗ seed oil content by up to 9%	↗ C16:0, C18:0, C18:1, C18:2, C18:3, C20:0, C22:0, C22:1	(Liu et al., 2022)
*FATB*	*A. thaliana*	*A. thaliana*	Down-regulation		↗ C16:1, C18:2; ↘C16:0, C18:0, C18:1, C18:3	(Bonaventure et al., 2003)
	*C. sativa*	*C. sativa*	Down-regulation		↗ C18:1, C18:3, C20:1, C22:1; ↘C16:0, C18:0, C20:0, C22:0	(Ozseyhan et al., 2018)
	*E. guineensis*	*A. thaliana*	Overexpression		↗ C16:0, C18:2, C18:3, C20:0; ↘ C18:0, C18:1	(Parveez et al., 2010)
			Down-regulation		↗ C18:2, C20:0; ↘ C18:0, C18:1, C18:3	(Parveez et al., 2010)
	*A. thaliana*	*A. thaliana*	Down-regulation		↗ C18:1, C18:2, C18:3; ↘ C16:0, C16:1, C18:0, C18:1, C20:0, C20:2, C22:0	(Belide et al., 2012)
	*G. max*	*G. max*	Down-regulation	↘ seed oil content by up to 10%	↗ C18:1; ↘ C16:0, C180, C18:2, C18:3	(Ma et al., 2021)
	*J. curcas*	*A. thaliana*	Overexpression	no significant difference	↗ C16:0, C18:0, C18:1, C18:2, C18:3, C20:0, C22:0; ↘ C22:1	(Liu et al., 2022)
	*D. butyracea*	*B. juncea*	Overexpression	↗ seed oil content by up to 5%	↗ C16:0, C18:1, C18:2; ↘ C22:1	(Sinha et al., 2007)
*FATB1 + LPAT2*	*C. viscosissima + C. viscosissima*	*T. arvense*	Overexpression		↗C8:0, C10:0, C12:0, C14:0, C16:0, C18:0; ↘ C18:1, C18:2, C18:3, C20:0, C20:1, C22:1, C22:2, C24:1	(Esfahanian et al., 2021)
*FATB1 + LPAT2 + DGAT1*	*C. viscosissima + C. viscosissima + C. avigera*	*T. arvense*	Overexpression		↗ C8:0, C10:0, C12:0, C14:0, C16:0, C18:0; ↘ C18:1, C18:2, C18:3, C20:0, C20:1, C22:1, C22:2, C24:1	(Esfahanian et al., 2021)
*FATB + LPAT*	*U. californica + C. nucifera*	*T. arvense*	Overexpression		↗ C8:0, C10:0, C12:0, C14:0, C16:0, C18:0; ↘ C18:1, C18:2, C18:3, C20:0, C20:1, C22:1, C22:2, C24:1	(Esfahanian et al., 2021)
*FATB2 + FatB2*	*C. avigera + C. hookeriana*	*T. arvense*	Overexpression		↗ C8:0, C10:0, C12:0, C14:0, C16:0, C18:0; ↘ C18:1, C18:2, C18:3, C20:0, C20:1, C22:1, C22:2, C24:1	(Esfahanian et al., 2021)
*FAX1*	*A. thaliana*	*A. thaliana*	Overexpression	↗ seed oil content by up to 34%		(Tian et al., 2018)
	*A. thaliana*	*C. sativa*	Overexpression	↗ seed oil content by 4%	↗ C18:0, C18:1, C18:2; ↘ C18:3, C20:1	(Cai et al., 2021)
	*B. napus*	*A. thaliana*	Overexpression	↗ seed oil content by 17%	↗ C18:1; ↘ C16:0, C20:0, C20:1	(Xiao et al., 2021)
*FAX2*	*A. thaliana*	*A. thaliana*	Overexpression	↗ seed oil content by 30%	↗ C18:2, C18:3	(Li et al., 2020)
			Overexpression	↗ seed oil content by up to 21%		(Tian et al., 2019)
			Down-regulation	↘ seed oil content by up to 18%		(Tian et al., 2019)
			Down-regulation		↘ C18:2	(Li et al., 2020)
*FAX4*	*A. thaliana*	*A. thaliana*	Overexpression	↗ seed oil content by 30%	↗ C18:2, C18:3	(Li et al., 2020)
			Down-regulation		↘ C20:1	(Li et al., 2020)
*FAX2 + FAX4*	*A. thaliana*	*A. thaliana*	Down-regulation	↘ seed oil content by up to 28%	↘ C18:3, C20:0	(Li et al., 2020)
*GPDH*	*G. max*	*G. max*	Overexpression	↗ seed oil content by 24%	↗ C18:1, C18:2, C18:3	(Zhao et al., 2021)
*LACS2*	*B. napus*	*B. napus*	Overexpression	↗ seed oil content by 8%	↗ C18:2, C20:0, C20:1, C22:0;↘ C18:3, C20:1	(Ding et al., 2020)
			Down-regulation	↘ seed oil content by 20%	no significant difference	(Ding et al., 2020)
*LACS4 + LACS9*	*A. thaliana*	*A. thaliana*	Down-regulation	↘ seed oil content by 5%	↗ C18:3; ↘ C18:2	(Jessen et al., 2015)
*MCAMT*	*A. thaliana*	*A. thaliana*	Overexpression	↗ seed oil content by 15-20%	↗ C18:3, C20:1; ↘ C18:2, C22:1	(Jung et al., 2019)
*NTT1*	*B. napus*	*B. napus*	Overexpression	↗ seed oil content by 5.5%		(Hong et al., 2022b
*NTT2*	*B. napus*	*B. napus*	Overexpression	↗ seed oil content by 8%	↗ C18:1, C18:3; ↘ C18:2	(Xia et al., 2022)
			Down-regulation	no significant difference	↗ C18:2; ↘ C18:1, C18:3	(Xia et al., 2022)
*PPT1*	*B. napus*	*B. napus*	Overexpression	↗ seed oil content by 3.3%	no significant difference	(Tang et al., 2022b)
			Down-regulation	↘ seed oil content by 9%	no significant difference	(Tang et al., 2022b)
*SAD*	*X. sorbifolia*	*A. thaliana*	Overexpression	↘ seed oil content by 8%	↗ C18:0, C20:0; ↘ C18:1, C18:2, C18:3, C20:1	(Zhao et al., 2015)
		*X. sorbifolia*	Down-regulation		↗ C16:0, C18:0; ↘ C18:1, C18:2, C18:3	(Zhao et al., 2015)
	*P. ostii*	*A. thaliana*	Overexpression	no significant difference	↗ C18:1, C18:3, C20:0, C20:1; ↘ C18:0	(Li et al., 2020a)
	*Z. mays*	*Z. mays*	Overexpression	↗ seed oil content by up to 5%	↘ C18:0	(Du et al., 2016)
			Down-regulation	↗ seed oil content by up to 10%	↗ C18:0; ↘ C18:1	(Du et al., 2016)
		*A. thaliana*	Overexpression	↘ seed oil content by up to 5%	↘ C18:0	(Du et al., 2016)
			Down-regulation	↗ seed oil content by up to 23%	↗ C18:0; ↘ C18:1	(Du et al., 2016)
	*R. communis*	*H. annuus*	Overexpression		↘ C18:0	(Rousselin et al., 2002)
*SAD + KASII_hairpin*	*C. sativa*	*C. sativa*	Overexpression		↗ C16:1 (omega-7), C18:1 (omega-7), C20:1 (omega-7); ↘ C18:0, C18:1, C18:2, C20:0, C20:1, C20:2, C22:0, C22:1	(Rodríguez-Rodríguez et al., 2021)

### Pull strategy

The pull strategy aims to ensure efficient assembly of FAs generated in the plastid into TG at the ER (**Figure 2**). Most of the metabolic engineering studies that attempted to optimize this step in seeds have focused on the over-expression of diacylglycerol acyltransferase (DGAT) (He et al., 2004; Kroon et al., 2006; Lung and Weselake, 2006; Shockey et al., 2006; Yu et al., 2006; Burgal et al., 2008; Misra et al., 2013; Wang et al., 2014; Liu et al., 2015; Savadi et al., 2015; Zhou et al., 2020; Chen et al., 2022) and phospholipid: diacylglycerol acyltransferase 1 (PDAT1) (Zhang et al., 2010; van Erp et al., 2011; Guan et al., 2015; Zhou et al., 2020) which commit acyl chains to storage TGs. Manipulation of other genes encoding for “pull” proteins, such as glycerol-3-phosphate dehydrogenases (GPDH), glycerol-3-phosphate acyltransferase (GPAT), and lysophosphatidic acid acyltransferase (LPAT), have also been reported to increase TG levels in seeds (Liu et al., 2015) (**Table 5**). Using the “pull” strategy, the combined over-expression of *DGAT*, *GPAT*, and *LPAT* was shown to be the most efficient to increase total seed oil content (+25%) (Shockey et al., 2019) (**Table 5**).

**Table 5:** List of “pull” genes manipulated to enhance oil yield and/or change FA composition in seeds.

Gene	Gene source	Target species	Strategy	Effect in total seed oil content	Effect in fatty Acid composition	References
*ABCA9*	*A. thaliana*	*A. thaliana*	Overexpression	↗ seed oil content by 24%	no significant difference	(Kim et al., 2013)
			Down-regulation	↘ seed oil content by 16%	↗ C18:2↘ C18:3	(Kim et al., 2013)
		*C. sativa*	Overexpression	↗ seed oil content by 22%		(Cai et al., 2021)
*ACP-desaturase*	*D. unguis-cati*	*A. thaliana*	Overexpression		↗ C16:0, C18:2, C18:3, C20:1, C22:1; ↘ C16:1, C18:0, C18:1, C20:0	(Bondaruk et al., 2007)
		*B. napus*	Overexpression		↗ C18:1; ↘ C16:0, C16:1, C18:0, C18:3	(Bondaruk et al., 2007)
*AAD*	*B. napus*	*B. napus*	Down-regulation	↘ seed oil content by up to 30%	↘ C18:1, C20:1	(Bryant et al., 2016a)
*ACBP*	*O. sativa*	*O. sativa*	Overexpression	↗ seed oil content by 10%	↗ C14:0, C16:0, C18:0, C18:1, C18:2, C18:3, C20:0, C22:0; ↘ C18:1	(Guo et al., 2019)
			Down-regulation	↘ seed oil content by 20%	↗ C16:1, C18:1, C20:1; ↘ C14:0	(Guo et al., 2019)
*DGAT*	*R. communis*	FAH transgenic *A. thaliana*	Overexpression		↗ C18:1-OH, C18:2-OH	(Burgal et al., 2008)
*DGAT + WRI1 + down-regulated SDP1*	*A. thaliana*	*A. thaliana*	Overexpression	↗ seed oil content by 16%	no significant difference	(van Erp et al., 2014)
*DGAT + WRI1*	*A. thaliana*	*G. max*	Over-expression	no significant difference	↗ C16:0, C18:0, C18:1, C18:3	(Arias et al., 2022)
*FAD*	*C. abyssinica*	*A. thaliana*	Down-regulation		↗ C18:1; ↘ C18:2, C20:1	(Li et al., 2016b)
*FAD+FAE*	*C. abyssinica*	*A. thaliana*	Down-regulation		↗ C18:1, C18:2; ↘ C18:3, C20:1	(Li et al., 2016b)
		*C. abyssinica*	Down-regulation		↗ C16:0, C18:0, C18:1, C20:1; ↘ C18:2, C18:3, C20:2, C22:0, C22:1, C24:0, C24:1	(Li et al., 2016b)
*FAD2*	*A. hypogaea*	*A. hypogaea*	Down-regulation		↗ C18:1; ↘ C18:2	(Yin et al., 2007)
	*G. max*	*G. max*	Down-regulation		↗ C18:1; ↘ C16:0, C18:2, C18:3	(Haun et al., 2014)
	*B. napus*	*B. napus*	Down-regulation	↘ seed oil content by up to 40%		(Chapman et al., 2008)
	*T. arvense*	*T. arvense*	Down-regulation		↗ C18:1, C20:1, C22:1; ↘ C16:0, C16:1, C18:2, C18:3	(Jarvis et al., 2021)
*FAD3*	*P. suffruticosa*	*A. thaliana*	Overexpression		↗ C16:0, C18:0, C18:3; ↘ C18:1, C18:2	(Yin et al., 2018)
	*A. thaliana*	*A. thaliana*	Down-regulation		↗ C18:2; ↘ C16:0, C18:1, C18:3	(Yin et al., 2018)
	*G. max*	*O. sativa*	Overexpression		↗ C18:3	(Anai et al., 2003)
*FAD6*	*P. irregulare*	*B. juncea*	Overexpression		↗ C18:3 (gamma); ↘ C18:0; C18:1, C18:2, C18:3	(Hong et al., 2002)
*FAD6 + FAD15*	*B. officinalis/A. thaliana*	*G. max*	Overexpression		↗ C18:3 (alpha and gamma), C18:4	(Eckert et al., 2006)
*FADX*	*P. granatum*	*A. thaliana*	Overexpression		↗ C18:1, punic acid; ↘ C16:0, C18:0, C18:2, C18:3	(Mietkiewska et al., 2014)
*FADX +FAD2*	*P. granatum*	*A. thaliana*	Overexpression		↗ punic acid; ↘ C16:0, C18:0, C18:2, C18:3	(Mietkiewska et al., 2014)
*FAE*	*C. abyssinica*	*A. thaliana*	Down-regulation		↗ C18:1; ↘ C18:2, C20:1	(Li et al., 2016b)
	*B. juncea*	*B. juncea*	Down-regulation	↗ seed oil content by up to 11%	↗ C16:0, C18:0, C18:1, C18:2; ↘ C18:3, C22:1	(Sinha et al., 2007)
	*T. majus*	*A. thaliana*	Overexpression		↗ C22:0, C22:1, C24:0; ↘ C18:0, C20:0, C20:1	(Mietkiewska et al., 2004)
*FAE1*	*E. hyemalis*	*B. carinata*	Overexpression		↗ C16:0, C18:0, C20:0, C20:1, C20:2, C20:3, C24:1; ↘ C18:1, C18:2, C18:3	(Meesapyodsuk et al., 2021)
	*A. thaliana*	*B. napus*	Overexpression	↗ seed oil content by up to 11%	↗ C22:1	(Katavic et al., 2000)
	*C. abyssinica*	*A. thaliana*	Overexpression		↗ C22:0, C22:1, C24:0, C24:1; ↘ C18:0, C18:1, C20:0, C20:1	(Mietkiewska et al., 2007)
		*B. carinata*	Overexpression		↗ C18:1, C22:0, C22:1, C24:0; ↘ C16:0, C18:0, C18:2, C18:3, C20:0, C20:1	(Mietkiewska et al., 2007)
	*T. arvense*	*T. arvense*	Down-regulation		↗ C16:0, C16:1, C18:0, C18:1, C18:2, C18:3; ↘ C20:1, C22:1, C24:1	(Jarvis et al., 2021)
*FAE1 + FAD2*	*T. arvense*	*T. arvense*	Down-regulation		↗ C18:0, C18:1; ↘ C16:0, C16:1, C18:2, C18:3, C20:1, C22:1, C24:1	(Jarvis et al., 2021)
*FAE1 + ROD1*	*T. arvense*	*T. arvense*	Down-regulation		↗ C16:0, C18:0, C18:1, C18:3; ↘ C16:1, C18:2, C20:1, C22:1, C24:1	(Jarvis et al., 2021)
*FAH*	*R. communis*	*A. thaliana*	Overexpression	↘ seed oil content by up to 50%	↗ HFA	(Bates et al., 2014)
		*A. thaliana*	Overexpression		↗ HFA	(Smith et al., 2003)
*FAH + (DGAT or PDAT)*	*R. communis*	*A. thaliana*	Overexpression	↘ seed oil content by up to 15%	↗ HFA	(Bates et al., 2014)
*FAH + FAE*	*P. fendleri/R. communis*	*C. sativa*	Overexpression		↗ C18:1-OH, C18:2-OH, C20:1-OH, C20:2-OH	(Snapp et al., 2014)
*KCS*	*C. graeca*	*A. thaliana*	Overexpression		↗ C22:1, C24:1, C26:1; ↘ C20:1	(Taylor et al., 2009)
		*B. carinata*	Overexpression		↗ C22:1, C24:1; ↘ C18:1, C18:2, C18:3	(Taylor et al., 2009)
*KCS+KCR+HCD+ECR*	*L. annua, A. thaliana*	*C. sativa*	Overexpression		↗ C16:0, C18:3, C22:0, C22:1, C24:0, C24:1; ↘ C18:0, C18:1, C18:2, C20:0, C20:1	(Huai et al., 2015)
*LPAAT2 + FAE1*	*E. hyemalis*	*B. carinata*	Overexpression	↗ seed oil content by up to 2%	↗ C16:0, C18:0, C20:2, C22:1, C22:2, C22:3, C24:1, C24:2, C24:3; ↘ C18:1, C20:1, C20:2	(Meesapyodsuk et al., 2021)
*LPAT+GPAT+DGAT*	*R. communis*	FAH transgenic *A. thaliana*	Overexpression	↗ seed oil content by up to 25%	↗ HFA	(Shockey et al., 2019)
*LPCAT*	*C. abyssinica*	*C. abyssinica*	Down-regulation		↗ C18:1, C20:1; ↘ C22:1	(Guan et al., 2015)
*PDAT*	*C. abyssinica*	*C. abyssinica*	Down-regulation		↗ C18:1; ↘ C18:2	(Guan et al., 2015)
	*R. communis*	FAH transgenic *A. thaliana*	Overexpression		↗ C18:1-OH, C18:2-OH	(van Erp et al., 2011)
*PDCT*	*C. abyssinica*	*C. abyssinica*	Down-regulation		↗ C18:1; ↘ C18:2	(Guan et al., 2015)
*PDCT+LPCAT*	*C. abyssinica*	*C. abyssinica*	Down-regulation		↗ C18:1, C20:1; ↘ C18:2, C22:1	(Guan et al., 2015)
*ROD1*	*T. arvense*	*T. arvense*	Down-regulation		↗ C16:0, C16:1, C18:1, C18:3, C20:1; ↘ C18:0, C24:1	(Jarvis et al., 2021)
*Δ6 and Δ5 desaturase + FAE*	*M. alpina* (fungus)	*G. max*	Overexpression		↗ C16:0, C18:0, C18:3 (gamma), C20:2, C20:3, C20:4; ↘ C18:1, C18:2, C18:3 (alpha)	(Chen et al., 2006)
*Δ6 desaturase*	*G. max*		Down-regulation			(Chen et al., 2006)
*Δ9 elongase*	*I. galbana*	*A. thaliana*	Overexpression		↗ C20:2, C20:3, C20:4, C20:5; ↘ C18:0, C18:1, C18:2, C18:3	(Petrie et al., 2012)
*Δ8* and *Δ5 desaturases*	*P. salina*					
*Δ9 elongase*	*I. galbana*	*B. napus*	Overexpression		↗ C16:0, C20:2, C20:3, C20:4, C20:5; ↘ C18:0, C18:1, C18:2, C18:3, C20:0	(Petrie et al., 2012)
*Δ8* and *Δ5 desaturases*	*P. salina*					
*Δ12-oleate hydroxylase*	*P. fendleri*	*A. thaliana*	Overexpression		↗ C18:1-OH, C18:2-OH, C20:1-OH, C20:2-OH	(Smith et al., 2000)

### Package strategy

As TGs accumulate within the ER phospholipid bilayer, the outer layer starts to expand into the cytosol, forming nascent LDs that bud off to form LDs (**Figure 2**). The “package” strategy aims to facilitate LD biogenesis and maximize droplet stability by proper coating. Proteomic analyses of seed LDs indicated that they contain 20–30 coat proteins which were found to be involved in LD formation and/or stability (Jolivet et al., 2004; Jolivet et al., 2009). These coat proteins might also help blocking the accessibility of the TG within the LD core to lipases allowing more LD formation. Recent evidence shows that seed-specific overexpression or down-regulation of some genes encoding these proteins increased LD size and/or number. Such proteins include lipid droplet-associated protein (LDAP) (Gidda et al., 2016), LDAP-interacting protein (LDIP) (Pyc et al., 2017), oleosin (Lu et al., 2006, Lu et al., 2018; Siloto et al., 2006; Shimada et al., 2008; Hu et al., 2009; Liu et al., 2013; Zhang et al., 2019), and seipins (Lunn et al., 2018) (**Table 6**). Interestingly, several studies also used mammalian “package” genes, such as fat-specific protein 27 (*FSP27*) and fat storage-inducing transmembrane protein 2 (*FIT2*) from mouse in *Arabidopsis*. These resulted not only in improvement of seed oil content but also stimulation of LD clustering and fusion (Cai et al., 2017; Price et al., 2020) (**Table 6**). Among all the mentioned “package” genes, over-expression of *seipin* in Arabidopsis gave the highest increase in seed oil content (+62%), which enhanced hydroxy-fatty acid (HFA) levels too (Lunn et al., 2018) (**Table 6**).

**Table 6:** List of “package” genes manipulated to enhance oil yield and/or change the FA composition in seeds.

Gene	Gene source	Target species	Strategy	Effect in total seed oil content	Effect in fatty Acid composition	References
*FIT2*	Mouse	*A. thaliana*	Overexpression	↗ seed oil content by up to 13%	↗ C16:0, C18:1; ↘ C20:2	(Cai et al., 2017)
*FSP27*	Mouse	*A. thaliana*	Overexpression	↗ seed oil content by up to 9%		(Price et al., 2020)
*LDAP*	*A. thaliana*	*A. thaliana*	Overexpression	no significant difference	↗ C16:0, C18:3; ↘ C18:1, C18:2, C20:1	(Gidda et al., 2016)
			Down-regulation	no significant difference	no significant difference	(Gidda et al., 2016)
*LDIP*	*A. thaliana*	*A. thaliana*	Down-regulation	↗ seed oil content by up to 22%	↗ C18:1, C18:2, C18:3, C20:1	(Pyc et al., 2017)
*Oleosin*	*C. tinctorius*	*A. thaliana*	Overexpression	↗ seed oil content by up to 30%		(Lu et al., 2018)
	*G. max*	*O. sativa*	Overexpression	↗ seed oil content by up to 46%	no significant difference	(Liu et al., 2013)
		*G. max*	Overexpression	↗ seed oil content by up to 10.6%	↗ C18:2	(Zhang et al., 2019)
*Seipin*	*A. thaliana*	*A. thaliana*	Overexpression	↗ seed oil content by up to 62%	↗ HFA	(Lunn et al., 2018)
			Overexpression	↗ seed oil content by up to 10%		(Cai et al., 2015)

### Protect strategy

Coat proteins can be hydrolyzed by endogenous proteases and TGs can be degraded into free FAs by various lipases to produce acetyl‐CoA *via* β‐oxidation particularly during seed germination and seedling growth (Pracharoenwattana and Smith, 2008; Li-Beisson et al., 2013; Borek et al., 2015) (**Figure 2**). Blocking the breakdowns of coat proteins and TGs (also known as “protect” strategy) is therefore an attractive strategy to increase oil content in seeds. The initial step in lipase-induced TG mobilization is the ubiquitination of the respective coat proteins, particularly oleosin and caleosin, and then subsequent digestion by the proteasome (Sorokin et al., 2009; Hsiao and Tzen, 2011; Deruyffelaere et al., 2015), which was extensively reviewed (Sorokin et al., 2009; Shao et al., 2019; Guzha et al., 2023). Important insights into the mechanism regulating the turnover of oleosins in plants has been recently published (Deruyffelaere et al., 2018; Kretzschmar et al., 2018). Two key components, PUX10 (a member of the plant ubiquitin regulatory X (UBX)-domain containing protein family) and CDC48A (the AAA ATPase, Cell Division Cycle 48) were identified. PUX10 localizes to LDs, binds to the ubiquitinated oleosins, and interacts with ubiquitin and CDC48A, respectively. As an adaptor, PUX10 recruits CDC48A to ubiquitinated oleosins, leading to dislocation of oleosins from LDs *via* the segregase activity of CDC48A (Deruyffelaere et al., 2018; Kretzschmar et al., 2018). In *Arabidopsis pux10* mutant seeds, PUX10 deficiency impaired the degradation of ubiquitinated oleosins from LDs. However, this did not change the total FA content in seeds of mutant genotypes in comparison to wild-type (Deruyffelaere et al., 2018; Kretzschmar et al., 2018) (**Table 7**). On the other hand, suppression of TG degradation to enhance FA content was achieved by down-regulating the genes that code for TG breakdown enzymes. Such genes are seed fatty acid reducer (*SFAR*) (Karunarathna et al., 2020), Gly-Asp-Ser-Leu (GDSL)-motif lipases (Ding et al., 2019), plastid lipase1 (*PLIP1*), and Sugar-dependent1 (*SDP1*) (Kelly et al., 2013; Kim et al., 2014; Kanai et al., 2019) (**Table 7**). Interestingly, recent studies reported that the over-expression of several genes with major role in TG degradation have a positive effect on seed oil content, which may seem counterintuitive. Such genes include patatin‐related phospholipase (*pPLAIIIδ*) (Li et al., 2013; Li et al., 2015a), and nonspecific phospholipase C6 (*NPC6*) (Cai et al., 2020) (**Table 7**). An intriguing question arising from these studies is how the over-expressed proteins increased lipid accumulation in seeds. One hypothesis is that phospholipase-mediated phospholipid turnover facilitates the movement of FAs from the plastid to the ER. Phosphatidylcholine (PC) is the most abundant class of phospholipids in plants and plays pivotal roles in TG production (Wang, 2005; Chapman and Ohlrogge, 2012; Karki et al., 2019). When hydrolyzed, PC can serve as a substrate for FA desaturation and also provides free FAs or DAG for TG synthesis (Mhaske et al., 2005; Lu et al., 2009; Zhang et al., 2010; Pokotylo et al., 2013). Therefore, over-expression of these phospholipases could enhance the acyl flux into ER and increase the overall levels of TGs in seeds. Among the genes tested as part of the “protect” strategy, it was shown that the down-regulation of *PLIP1* in Arabidopsis gave the highest increase in seed oil content (+45%) (Wang et al., 2017) (**Table 7**).

**Table 7:** List of “protect” genes used to enhance oil yield and/or change the FA composition in seeds.

Gene	Gene source	Target species	Strategy	Effect in total seed oil content	Effect in fatty Acid composition	References	
*SFAR*	*B. napus*	*B. napus*	Down-regulation	↗ seed oil by 14-28%	↗ C18:3, C20:0, C20:1, C22:0, C22:1; ↘ C16:0, C16:1	(Karunarathna et al., 2020)	
*GDSL1*	*A. thaliana*	*B. napus*	Overexpression	↘ seed oil by 13%	↗ C18:2, C18:3; ↘ C18:1, C20:0, C20:1	(Ding et al., 2019)	
	*B. napus*	*B. napus*	Overexpression	↘ seed oil by 13%	↗ C18:2, C18:3; ↘ C18:1, C20:0, C20:1	(Ding et al., 2019)	
	*B. napus*	*B. napus*	Down-regulation	↗ seed oil by 12%	↗ C18:1; ↘ C18:2, C18:3	(Ding et al., 2019)	
*NCP6*	*A. thaliana*	*A. thaliana*	Overexpression	↗ seed oil by 6-8%	↗ C18:3, C20:0, C20:1; ↘ C16:0 and C18:0	(Cai et al., 2020)	
	*C. sativa*	*C. sativa*	Overexpression	↗ seed oil by 6.3%	↗ C18:3; ↘ C16:0 and C18:0	(Cai et al., 2020)	
*pPLAIIIδ*	*C. sativa*	*C. sativa*	Overexpression	↗ seed oil by 14%	↗ C20:1; ↘ C18:1	(Li et al., 2015a)	
*PLIP1*	*A. thaliana*	*A. thaliana*	Overexpression	↘ seed oil by 25%	↗ C18:3; ↘ C18:1, C18:2	(Wang et al., 2017)
			Down-regulation	↗ seed oil by 45%	↗ C18:1, C18:3, C22:1; ↘ C20:0	(Wang et al., 2017)
*PUX10*	*A. thaliana*	*A. thaliana*	Down-regulation	no significant difference	↘ C18:2; ↗ C18:3	(Deruyffelaere et al., 2018)	
			Down-regulation	no significant difference	no significant difference	(Kretzschmar et al., 2018)	
*SDP1*	*J. curcas*	*A. thaliana*	Down-regulation	↗ seed oil by 13%	↗ C20:1; ↘ C18:1	(Kim et al., 2014)	
	*G. max*	*G. max*	Down-regulation	↗ seed oil by 30%	↗ C18:1; ↘ C18:2	(Kanai et al., 2019)	
	*B. napus*	*B. napus*	Down-regulation	↗ seed oil by 8%	no significant difference	(Kelly et al., 2013)	

### Modifying FA composition

Metabolic engineering strategies have been used to produce novel high-value FAs or to improve their synthesis in seeds of established crops and other model species (**Tables 3** **-** **7**). For instance, over-expression and down-regulation of desaturases, such as acyl-acyl carrier protein desaturase (AAD), fatty acid desaturases (FADs), and stearoyl-acyl carrier protein desaturase (SAD), and FAT, and/or down-regulation of fatty acid elongase (FAE) genes were attempted to divert the carbon flux towards the synthesis of beneficial FAs required for human health and nutrition, such as gamma-linolenic acid (C18:3), alpha-linolenic acid (C18:3), docosadienoic acid (C22:2), docosatrienoic acid (C22:3) (Hong et al., 2002; Anai et al., 2003; Eckert et al., 2006; Huai et al., 2015; Yin et al., 2018; Meesapyodsuk et al., 2021), and oils enriched in omega-7 monounsaturated FAs such as palmitoleic acid (C16:1) and its elongation products vaccenic acid (C18:1) and paullinic acid (C20:1) (Nguyen et al., 2015; Ettaki et al., 2018; Rodríguez-Rodríguez et al., 2021) (**Tables 4** **-** **5**). Also, several oilseed plants produce industrially-relevant FAs, such as erucic acid (C22:1), lesquerolic acid (C20:1-OH), and nervonic acid (C24:1), providing renewable alternatives to petrochemicals for the manufacture of lubricants, coatings, or polymers. However, most plants producing these FAs usually have undesirable traits and are not economically viable crops. It is hence important to improve the production of these valuable FAs in other established crops *via* metabolic engineering technology. One example is the improvement of the production of erucic and nervonic acids. VLCFAs, such as erucic acid (C22:1) and nervonic acid (C24:1), are important in plastic, cosmetic, nylon, and lubricant industries (Mastebroek and Marvin, 2000). Nervonic acid has only been found in the seed oils of a few known plants, and among these, only *Lunaria annua* has been considered as a niche crop for future development. However, this plant is a biennial with highly variable seed yields (800–2,000 kg/ha) and has a major problem of seed shattering, making it an uneconomical source of nervonic acid (Mastebroek and Marvin, 2000). Consequently, there is a high demanded to improve the production of this valuable FA in other oilseed crops *via* metabolic engineering technology (Taylor et al., 2009). Over-expression of 3-ketoacyl-CoA synthase (KCS) and FAE genes were shown to increase the production of erucic and nervonic acids in *Brassica napus*, *Camelina sativa*, and *Arabidopsis thaliana* (Katavic et al., 2000; Mietkiewska et al., 2004; Taylor et al., 2009; Huai et al., 2015) (**Table 5**). Another example is the production of HFAs in a toxin free oilseed crop to replace castor oil as a renewable source for numerous industrial applications. This was achieved through over-expression of fatty acid hydroxylase and elongase genes, and also acyltransferase genes from species producing HFAs, such as castor bean (*Ricinus communis*) and lesquerella (*Physaria fendleri*), in the seeds of the model species *Arabidopsis* and in the industrial oilseed crop *Camelina* (Smith et al., 2003; van Erp et al., 2011; Bates et al., 2014; Snapp et al., 2014; Shockey et al., 2019) (**Table 5**).

### Combining multiple strategies

To further increase the TG levels in seed oil, several studies have combined genes from different strategies. It is important to note that this combinatorial strategy has been mostly used to increase TG content in non-seed organs, such as leaves and roots (Slocombe et al., 2009; Fan et al., 2013; Kelly et al., 2013; Vanhercke et al., 2013; Winichayakul et al., 2013), and were extensively reviewed (Cahoon et al., 2007; Taylor et al., 2011; Xu et al., 2018; Vanhercke et al., 2019; Singh et al., 2021). However, it was shown that seed-specific overexpression of *AtWRI1* (“push”) and *AtDGAT1* (“pull”) combined with suppression of the triacylglycerol lipase SUGAR- DEPENDENT1 (*AtSDP1*) (“protect”) resulted in a higher seed oil content than manipulation of each gene individually (van Erp et al., 2014a) (**Table 5**). Similarly, seed specific over-expression of *Seipin* (“package”) in transgenic *Arabidopsis* expressing *R. communis* fatty acid hydroxylase (FAH) (“pull”) not only increased the total oil content but also increased the HFA composition in seeds (Lunn et al., 2018) (**Table 6**). However, this combinatorial strategy is not guaranteed to succeed: several attempts did not increase TG in seeds, such as the over-expression of *WRI1* (“push”) and *DGAT1*(“pull”) from *Arabidopsis* in soybean, suggesting that some species put in place counteracting processes to keep oil content stable (Arias et al., 2022) (**Table 5**). On the other hand, combining multiple strategies has also been successful to modify FA composition in seeds (**Tables 4** **-** **5**). For instance, co-expression of *Cuphea viscosissima FATB* (“Push”) and *LPAT* (“pull”) with *Cuphea avigera DGAT* (“pull”) increased the accumulation of TGs rich in medium-chain FAs (C6-C14) in pennycress for industrial, jet fuel and improved biodiesel applications (Esfahanian et al., 2021) (**Table 4**). Another example is the co-expression of *Δ6 and Δ5 desaturases* (“pull”) and *FAE* (“pull”) from a fungal species *(Mortierella alpinia)* in soybean, which caused the seed to accumulate novel FAs that are not naturally produced in soybean: γ-linolenic acid (GLA), eicosa-8, 11-dienoic acid (EDA), dihomo-γ-linolenic acid (DGLA), and arachidonic acid (C20:4), which are important for human health (Chen et al., 2006) (**Table 5**).

## Perspectives and challenges

The demands in seed oils for food and feed is rapidly increasing with growing population, urbanization, and industrialization. Enhancing oil production and improving FA composition in oilseed crops through metabolic engineering is a promising venue to meet these demands; the challenge is in identifying the right target(s). ^13^C-MFA has played a pivotal role in advancing our understanding of plant FAS at the systems level. The flux maps of primary metabolism that have emerged from MFA provide information on regulatory steps and pathways to be assessed within the context of the whole network, leading to the identification of candidate genes to be engineered for oil improvement. Current advances in mass spectrometry imaging techniques coupled with ^13^C-isotopic pulse labeling would also allow to assess spatial and temporal resolution of metabolic fluxes (Romsdahl et al., 2021). Recent discoveries of a number of genes involved in the “push, pull, package, and protect” steps of oil synthesis have enabled successful engineering of oil content and composition in different crops. Effective strategies combined the overexpression of TFs that upregulate FAS and genes involved in TG assembly, combined with the downregulation of TG catabolic enzymes. The challenge is that a given strategy may work in a species but not in others: as demonstrated by ^13^C-MFA studies, developing embryos from different species use different pathways, sometimes even non-conventional reactions, for FAS. Finally, the comprehensive understanding of multi-”omics” technologies and advanced genome-editing capabilities offer the possibility of rapid assembly and introduction of multiple candidate genes for further improvements in seed oil quality. Popular genome-editing tools, such as CRISPR/Cas9, could be used to remove or minimize metabolic competition while directing metabolic flux toward TG biosynthesis, or to edit specific amino acids in oil biosynthesis enzymes to improve/modify enzymatic activities (Park and Kim, 2022).

## Author contributions

AA conceived the idea. JS and UY drafted the manuscript and designed the figures. AA reviewed and edited the manuscript. All authors contributed to the article and approved the submitted version.
